# Breast screening with magnetic resonance imaging in high-risk women

**DOI:** 10.1186/bcr3306

**Published:** 2012-11-09

**Authors:** L McLean, F Douglas, N Forester, CE Holmes

**Affiliations:** 1 Breast Screening Unit, Newcastle, UK; 2 Department of Genetics, Newcastle, UK

## Introduction

The Cancer Reform Strategy [1] and NICE guidelines [2] highlighted the need to identify women at increased genetic risk of breast cancer. Following this, a magnetic resonance imaging (MRI) screening service was established in Newcastle.

## Methods

Women were identified from the Regional Genetic Centre database, following assessment by a consultant geneticist as high risk according to the NICE guidelines [2]. Screening comprised annual MRI ± mammography, depending on their age, following the standards set by the NHSBSP [3]. All investigations were double-read by NHSBSP-compliant radiologists.

## Results

A total of 142 women underwent 311 screening episodes between January 2008 and June 2012. There were 28 recalls (9%) for second-look ultrasound in 24 women. Thirteen lesions had a core biopsy, from which six (1.9%) malignancies were identified of varying histological type, size and grade. Seven lesions were benign. No ultrasound abnormality was found in the remaining 15 lesions. No interval cancers have developed.

## Conclusion

In our unit, high-risk MRI screening is effective and efficient, with a cancer detection rate of 0.2 per 1,000 women screened and no false negative screens to date, with an acceptable recall rate for second-look ultrasound (within the standard set under the NHSBSP guidelines). This has been possible due to the close liaison between the departments of Genetics and Breast Screening.
